# Alcohol-related mortality and all-cause mortality following bereavement in two successive generations

**DOI:** 10.1371/journal.pone.0243290

**Published:** 2020-12-10

**Authors:** David Teye Doku, Subas Neupane, Henrik Dobewall, Arja Rimpelä

**Affiliations:** 1 Department of Population and Health, University of Cape Coast, Cape Coast, Ghana; 2 Faculty of Social Sciences, Tampere University, Health Sciences, Tampere, Finland; 3 PERLA (Tampere Centre for Childhood, Youth and Family Research), Tampere University, Tampere, Finland; 4 Department of Adolescent Psychiatry, Tampere University Hospital, Pitkäniemi, Finland; Leibniz Institute for Prevention Research and Epidemiology BIPS, GERMANY

## Abstract

**Background and aim:**

Bereavement affects the health of the bereaved both emotionally and physically. Bereavement resulting from alcohol-related death of the previous generation (parents-first generation) may increase the risk of alcohol abuse and consequently alcohol-related mortality as well as all-cause mortality in the next generation (offspring-second generation). Furthermore, these associations can be bi-directional. However, there is no conclusive evidence of these effects, and studies exploring these intergenerational effects are rare. This study investigates these associations.

**Methods:**

A longitudinal data were constructed by linking participants from the Adolescent Health and Lifestyle Surveys (AHLS) from 1979 to 1997 with census and registry-based data from Statistics Finland containing the socioeconomic status of the survey participants and their parents (N = 78610) to investigate these associations. Multivariate Cox proportional hazards models were used to calculate hazard ratios with 95% confidence intervals to determine the effect of bereavement with alcohol-related mortality and all-cause mortality.

**Results:**

The findings suggest that bereavement following the death of an offspring increases the risk of both alcohol-related and all-cause mortality among both parents. The magnitude of the risk of mortality following the death of an offspring is higher for mothers than for fathers. There were no clear associations of a parent’s death with an offspring’s alcohol-related or all-cause mortality. However, generally, a father’s death seems to be protective of the risk of mortality among the offspring while a mother’s alcohol-related death slightly increased the risk of alcohol-related mortality among their offspring.

**Conclusions:**

These findings emphasise the role of bereavement, particularly resulting from the death of an offspring, on alcohol-related and all-cause mortality and therefore inequalities in mortality. Furthermore, the findings highlighting the need for alcohol abuse intervention and emotional support for bereaved persons following the death of an offspring.

## Introduction

Bereavement is a critical event, which affects the health of the bereaved both emotionally and physically [1]. Parental death is a stressful life-event for offspring, but there is no conclusive evidence of the effect of a parent’s death on an offspring’s life. Some studies have reported that the risk of depression, physical illness, suicides [2] and poorer school performance [3] are associated with parental death, while others found no association [4].

The death of an offspring is associated with a number of adverse consequences for surviving parents, including psychiatric hospitalization [5] and mortality [6,7]. However, we found no earlier studies that have studied the association of parental alcohol-related mortality with the alcohol-related death of an offspring and vice versa. Evidence from systematic review of prospective cohort studies shows that parental drinking is associated with drinking in their offspring [8]. We posit that bereavement resulting from alcohol-related death of the previous generation (parents-first generation) may increase the risk of alcohol abuse and consequently alcohol-related mortality as well as all-cause mortality in the next generation (offspring-second generation). We further posit that these associations are bi-directional such that bereavement resulting from alcohol-related death of the second generation may also increase the risk of alcohol abuse and hence alcohol-related mortality as well as all-cause mortality in the first generation. We aim to test the above postulations.

## Methods

### Study design and data

We constructed longitudinal data by linking participants from the Adolescent Health and Lifestyle Surveys (AHLS) from 1979 to 1997 with census (collected every fifth year until 1995) and registry-based (yearly information from 2000 onwards) data from Statistics Finland containing the socioeconomic status of the survey participants and their parents (N = 78610). In this study, we refer to the parents and the adolescents (offspring) as the first and second generations, respectively. The AHLS surveys were conducted among nationally representative samples of 12-, 14-, 16-, and 18-year-olds drawn from the Finnish Population Register [9]. The AHLS has been conducted biennially by postal and later on also by internet surveys since 1977. Participation in the AHLS has been voluntary, and no parental consent has been demanded. Statistics Finland linked the survey data to the registries in accordance with a contract specifying the rights and duties of both parties and its Institutional Review Board and the Data Protection Ombudsman approved the study protocol. Identification of the study participants was withheld from the investigators at all stages of the study. The Joint Commission on Ethics of the University of Turku and the Turku University Hospital stated that no human rights were violated in the research protocol and approved it.

Follow-up data were obtained from the Finnish Official Cause-of-Death Register, which contains statistics on all residents in Finland. For the second generation (offspring), the follow-up started on 30^th^ April each survey year, and ended on 31^st^ December 2009, or when the participant died while that for the first generation (fathers and mothers) started on 1^st^ January 1970 and ended 31^st^ December 2009.

The main outcomes were alcohol-related mortality and all-cause mortality. These were defined by the date of death (month, year) and cause of death. Alcohol-related death refers to death resulting directly from alcohol related diseases such as liver diseases. It does not include deaths from alcohol-related injuries such as deaths from alcohol-related motor traffic accidents. For the second generation, we obtained information on age, sex, and date of birth from both the AHLS and the Finnish population register while the latter provided the corresponding information for the first generation. Deaths from other causes other alcohol-related were coded as “other causes of death”. Information on the date of death and cause of death for both the first and second generations were obtained from the Finnish official cause-of-death register.

Socioeconomic status of the parents followed the classifications of Statistics Finland [10]. We formed variables that described the parents’ socioeconomic circumstances nearest to the age when the adolescent was 15 years old, corresponding to the mean age of the participants. Socioeconomic measures within five years of the offspring’s 15^th^ birthday were used since the exact 15 years was not available for everybody because the early censuses were collected every fifth year. *Socioeconomic status* (SES) was classified as upper white-collar, lower white-collar, blue-collar, agricultural entrepreneur, other (pensioners, students, those in military service) and unknown.

### Statistical analysis

We used Cox proportional hazards models to calculate hazard ratios (HR) with 95% confidence intervals (CIs) to determine the associations of the predictor variables with mortality. First, we examined the associations using a sex-adjusted models. Second, we used multivariable models adjusted for sex, and socioeconomic status of each parent separately for the models which predicted father’s and mother’s mortality following the death of their offspring while for the models which predicted an offspring’s mortality following the death of a father or a mother, we adjusted for sex, and father’s and mother’s socioeconomic status together. We tested the proportionality hazard assumption of the Cox model using log-log survival curves and unscaled and scaled Schoenfeld residuals [11]. Although some of the models did not meet the proportionality assumption, the socioeconomic predictors were retained because of their relevance in the light of previous literature [6,11]. STATA version 14 statistical software (StataCorp, College Station, TX, USA) was used for all analyses.

## Results

### Characteristics of the study population

The mean follow-up time for offspring was of 20.55 years (95% confidence interval (CI) 20.51–20.59) and the total follow-up time was 1, 615, 277 person-years. Fathers had a total follow-up time of 2, 931, 073 person-years with a mean of 37.29 years (95% CI 37.24–37.33), while mothers had a total follow-up time of 3, 054, 835 person-years with a mean of 38.86 years (95% CI 38.83–38.89). The total number of all-cause of death among offspring was 1, 286 (1.64%) of which 293, representing 22.78% of all deaths, were alcohol-related. Of fathers, the all-cause death was 15, 076 (19.18%), and 1, 521 of this was alcohol-related, representing 10.09% of all-cause of death among them. Correspondingly, 6120 (7.79%) of mothers died of all-cause of which 282, representing 4.61%, were alcohol-related. Males constitute 51.08% (N = 40154) of the offspring and the proportion of females was 38, 456 (48.92%). The proportion of the deaths of all-cause and alcohol-related deaths among the males were 75.97% (N = 977) and 88.74% (N = 260), respectively. The mean age at death for the offspring was 28.74 years (95% CI 28.32–29.17). The mean age at death for fathers and mothers were 58.28 years (95% CI 58.08–58.48) and 58.59 years (95% CI 58.27–58.91), respectively. The distribution of alcohol-related deaths and deaths of other causes of the first generation (parents) and second generations (offspring) are presented in Table 1.

**Table 1:** Distribution of alcohol-related deaths and deaths of other causes of the first generation (parents) and second generations (offspring).

First generation	N (%)
**Father**	
Upper white-collar	19085 (24.28)
Lower white-collar	11485 (14.61)
Blue-collar	26148 (33.26)
Agricultural entrepreneur	6324 (8.04)
Others	11571 (15.51)
Missing	3997 (5.08)
**Total**	**78610 (100)**
**Father died before the child**	
No (alive)	63708 (81.04)
Alcohol related death	1497 (1.90)
Other causes of death	13405 (17.05)
**Total**	**78610 (100)**
**Mother**	
Upper white-collar	12280 (15.62)
Lower white-collar	29716 (37.80)
Blue-collar	18869 (24.00)
Agricultural entrepreneur	5738 (7.30)
Others	11242 (14.30)
Missing	765 (0.97)
**Total**	**78610 (100)**
**Mother died before the offspring**	
No (alive)	72590 (92.34)
Alcohol-related deaths	278 (0.35)
Other causes of death	5742 (7.30)
**Total**	**78610 (100)**
**Second generation**	
**Sex**	
Male	40154 (51.08)
Female	38456 (48.92)
**Total**	**78610 (100)**
**Offspring died before father**	
No (alive)	78361 (99.68)
Alcohol-related deaths	50 (0.06)
Other causes of death	199 (0.25)
**Total**	**78610 (100)**
**Offspring died before mother**	
No (alive)	78513 (99.88)
Alcohol-related deaths	27 (0.03)
Other causes of death	70 (0.09)
**Total**	**78610 (100)**

The associations of bereavement following the death of a father or a mother with a offspring’s alcohol-related death and death due to other causes are presented in Table 2.

**Table 2:** Association of alcohol-related deaths and deaths of other causes of the first generation (parents) with the second generation’s (offspring) alcohol-related deaths and deaths of all-causes in sex-adjusted and multivariate models.

First generation		Second generation (offspring)			
**Father**		**Sex-adjusted models**		**Multivariate models**	
	**Father died before the child**	**Alcohol-related deaths**	**All causes of death**	**Alcohol-related deaths**	**All causes of death**
		**HR, 95% CI**	**HR, 95% CI**	**HR, 95% CI**	**HR, 95% CI**
	No (alive)	1.00	1.00	1.00	1.00
	Alcohol-related deaths	0.98 (0.43–2.21)	1.22 (0.86–1.73)	0.56 (0.39–0.81)	1.01 (0.68–1.50)
	Other causes of death	0.65 (0.47–0.91)	0.73 (0.63–0.85)	0.56 (0.39–0.81)	0.62 (0.52–0.73)
**Mother**					
	**Mother died before the offspring**				
	No (alive)	1.00	1.00	1.00	1.00
	Alcohol-related deaths	0.91 (0.13–6.50)	1.22 (0.55–2.72)	0.97 (0.13–6.90)	1.14 (0.47–2.75)
	Other causes of death	0.93 (0.62–1.40)	0.70 (0.56–0.87)	0.89 (0.56–1.39)	0.64 (0.50–0.82)

In sex-adjusted models, father’s death due to other causes was negatively associated with a offspring’s alcohol-related mortality (HR = 0.65 (CI 0.47–0.91)), compared to those whose parents were alive. The hazard ratios also suggested a lower risk of alcohol-related mortality among the offspring when a parent (particularly a father) died independent of whether the death was alcohol-related (father HR = 0.80 (CI 0.33–1.94)) or due to other causes (father HR = 0.56 (CI 0.39–0.81)), although the estimates were not statistically significant. Also, death of a father or a mother due to other causes was associated with reduced risk of all-cause mortality of an offspring. On the other hand, in the sex-adjusted models, there were indications of elevated risks, with a father’s (HR = 1.22 (CI 0.86–1.73) and a mother’s (HR = 1.22 (CI 0.55–2.72) alcohol-related death potentially being associated with higher risk of an offspring’s all-cause mortality, although the confidence intervals overlap which could be due to small number of cases. In the multivariable models, the magnitudes of most of the associations of the death of a mother or father with an offspring’s mortality slightly increased, with the exception of the association between a mother’s and an offspring’s alcohol-related death which increased yet remained non-significant. Also, the associations of a mother’s alcohol-related death with an offspring’s all-cause mortality remained nearly the same.

Table 2 shows the alcohol-related death and death by other causes of the first generation (parents) and their associations with second generation’s (offspring) alcohol-related mortality and all-cause mortality. An offspring’s alcohol-related death or death of other causes was associated with higher risk of alcohol-related mortality and all-cause mortality in both fathers and mothers. The excess risk of mortality following the death of an offspring was higher for mothers than for fathers for both outcomes, although the CIs overlapped. Furthermore, in a multivariable model, the associations of an offspring’s death with father’s and mother’s mortality attenuated but the changes in the CIs were small (Table 3).

**Table 3:** Association of alcohol-related deaths and deaths of other causes of the second generation (offspring) with the first generation’s (parents) alcohol-related deaths and deaths of all-causes in sex-adjusted and multivariate models.

		First generation (parents)			
		**Father**			
		**Sex-adjusted models**		**Multivariate models**	
**Second generation (offspring)**		**Alcohol-related deaths**	**All causes of death**	**Alcohol-related deaths**	**All causes of death**
		**HR, 95% CI**	**HR, 95% CI**	**HR, 95% CI**	**HR, 95% CI**
	**Child died before parent**				
	No (alive)	1.00	1.00	1.00	1.00
	Alcohol-related deaths	31.15 (13.94–69.62)	18.32 (13.87–24.20)	26.80 (11.99–59.89)	16.84 (12.75–22.26)
	Other causes of death	27.18 (18.39–40.18)	16.48 (14.31–18.97)	25.46 (17.22–37.64)	15.96 (13.87–18.38)
		**Mother**			
	**Child died before parent**				
	No (alive)	1.00	1.00	1.00	1.00
	Alcohol-related deaths	34.83 (4.86–249.81)	38.86 (26.58–56.80)	30.98 (4.32–222.26)	36.65 (25.07–53.58)
	Other causes of death	62.91(25.77–153.55)	35.84 (28.27–45.42)	54.49 (22.31–133.04)	33.28 (26.25–42.19)

## Discussion

Our findings support the proposition that bereavement resulting from alcohol-related death of the second generation may also increase the risk of alcohol abuse and consequently alcohol-related mortality as well as all-cause mortality in the first generation. We expected that bereavement resulting from alcohol-related death of the previous generation (parents-first generation) may increase the risk of alcohol abuse and consequently alcohol-related mortality as well as all-cause mortality in the next generation (offspring-second generation). However, there were no clear associations of a parent’s death with the offspring’s mortality for alcohol-related or all-cause deaths. Generally, a father’s death appears to be protective of the risk of mortality among the offspring while a mother’s alcohol death tends to slightly increase the risk of alcohol-related mortality. Furthermore, this study found an increased risk of alcohol-related and all-cause mortality in fathers and mothers whose offspring had died either of alcohol-related or other causes. Our findings suggest that the excess risk of mortality following the death of an offspring might be higher for mothers than for fathers.

We found associations of bereavement after the death of an offspring with a higher risk of alcohol-related mortality among both fathers and mothers. The death of a loved one is a known stressful life event with varied implications depending on the relationship between the deceased and the bereaved [1]. Naturally, older persons are expected to die before younger ones, which means that parents are expected to die before their offspring. In most Western countries in particular, because of the advancement in health care, the death of a parent normally precedes the death of an offspring. Therefore, the death of an offspring is premature and unexpected and can lead to prolong acute grief [1]. Bereavement resulting from the death of an offspring is more intense compared with bereavement following the death of other loved ones, including a parent or a spouse [12,13]. Consequently, the death of an offspring can have adverse effect on the health of the parents including mental impairment, risk of cancer, cardiac events, insomnia, depression, anxiety, suicide ideation and attempt and higher risk of mortality [1]. One explanation of the association of bereavement with mortality is through indirect pathway of behaviour change such as alcohol abuse, smoking and poor diet habits as inadequate ways of dealing with the grief [1,14]. Although there was evidence of these mechanisms in our studies, this was not conclusive because the findings were not statistically significant.

Additionally, we found a higher risk of all-cause mortality among both fathers and mothers following the loss of an offspring consistent with other studies, which reported an increased risk of all-cause mortality among parents following the death of an offspring [6,15]. Aside from the indirect pathway of the effect of bereavement on health through health behaviour, a direct mechanism through psychobiological pathways involving severe shock among the bereaved has been suggested as explaining the higher risk of mortality among them [16]. It is argued that the risk of mortality because of the experience of unexpected bereavement such as the loss of an offspring is independent of one’s socioeconomic status or good health at baseline [17]. Studies have shown that the body’s response to bereavement leads to increased cortisol dysregulation cellular inflammation and deterioration in the immune system [1,18,19]. Furthermore, the excess risk of mortality following the death of an offspring was higher for mothers than for fathers as found in other previous studies [6,15]. Stronger attachment of offspring to their mothers and generally, greater mother’s involvement in their offspring could account for this finding.

Generally, we did not find consistent evidence supporting a risk of alcohol-related or all-cause mortality of an offspring following the death of a parent. However, overall, a father’s death seems to be protective of the risk of mortality among the offspring while a mother’s alcohol-related death slightly increased the risk of alcohol-related mortality, albeit not statistically significant. Also, a mother’s death resulting from other causes decreased the risk of both alcohol-related mortality and all-cause mortality of the offspring. Apart from the negative effect on health, it has been argued that bereavement can also have a positive effect on health, including maturity, improved coping skills and appreciation for life [20]. This support our finding of the protective effect of a father’s death with the risk of mortality among the offspring.

We used large samples and nationally representative data to investigate the effect of bereavement on the risk of alcohol-related mortality and all-cause mortality in two successive generations in both directions. Our study is, however, limited by the relatively few cases of the outcomes, particularly alcohol-related deaths among offsprings and mothers in the sample.

In conclusion, our findings suggest a higher risk of both alcohol-related mortality and all-cause mortality among bereaved persons following the death of an offspring. Overall, bereavement following the death of an offspring tends to have effect on alcohol-related mortality than all-cause mortality. Furthermore, the magnitude of the effect of bereavement on mortality was stronger for mothers than for fathers. Our findings underscore the need for alcohol abuse intervention and emotional support for bereaved persons following the death of an offspring.
